# Efficacy and safety of sini powder combined with Xiaoxianxiong decoction for adolescent depression with liver qi stagnation and phlegm-heat obstruction syndrome: a randomized controlled trial

**DOI:** 10.3389/fped.2026.1787781

**Published:** 2026-06-12

**Authors:** Ruiyu Lin, Yangzhen Liu, Juncheng Li, Jia Gao, Liting He, Shanying Huang

**Affiliations:** 1 The Third Hospital of Mianyang, Mianyang, Sichuan, China; 2 Sichuan Mental Health Center, Mianyang, Sichuan, China; 3 The Affiliated Mianyang Hospital of Chongqing Medical University, Mianyang, Sichuan, China; 4 Department of Pediatrics, Youxian District Hospital of Traditional Chinese Medicine (Youxian Branch of The Third Hospital of Mianyang), Mianyang, Sichuan, China; 5 The Third Hospital of Mianyang, Mianyang, Sichuan, China

**Keywords:** add-on therapy, adolescent depression, Chinese medicinal compound, clinical efficacy, liver qi stagnation, phlegm-heat blocking the middle energizer, randomized controlled trial, safety

## Abstract

**Objective:**

To assess efficacy and safety of Sini San combined with Xiaoxianxiong Tang as add-on therapy for adolescents (12–18 years) with liver qi stagnation and phlegm-heat depression, and to provide preliminary evidence for TCM syndrome-based treatment.

**Methods:**

Sixty adolescents (12–18 years) with moderate depression (DSM-5 criteria) meeting TCM syndrome standards were recruited from Youxian Branch of Mianyang Third Hospital (Jan 2024–Dec 2025). Sample size was calculated via G*Power 3.1 (effect size=0.8, *α*=0.05, *β*=0.2). A conservative effect size of 0.6 for TCM add-on trials in adolescent depression required 44 participants per group. Our sample size was calculated using the commonly adopted large effect size (0.8) for such trials, which was adequate to detect the predefined clinically meaningful effect. Participants were randomly assigned to TCM decoction+psychological counseling (*n* = 30) or psychological counseling alone (*n* = 30) with allocation concealment, receiving 8-week intervention.Intention-to-treat (ITT) analysis with last observation carried forward (LOCF) for missing data was used. All outcomes were assessed by independent blinded evaluators. Primary outcomes: CDRS-R and TCM syndrome scores; secondary outcomes: PedsQL score, clinical effective rate, adverse events. Cohen's d and 95% CIs were calculated with Bonferroni correction for multiple comparisons.

**Results:**

Baseline characteristics were comparable between groups (all *P* > 0.05). After 4 and 8 weeks, both groups showed improved outcomes (all *P* < 0.001). At 8 weeks, the study group had lower CDRS-R (13.02 ± 3.25 vs. 21.85 ± 3.56, t = 10.23, *P* < 0.001, Cohen's d = 2.64, 95%CI:1.72–3.56), lower TCM syndrome scores (3.21 ± 1.35 vs. 6.98 ± 1.62, t = 9.87, *P* < 0.001, Cohen's d = 2.52, 95%CI:1.61–3.43), higher PedsQL scores (79.35 ± 7.61 vs. 66.89 ± 7.43, t = 6.54, *P* < 0.001, Cohen's d = 1.68, 95%CI:0.92–2.44), and higher clinical effective rate (90.0% vs. 63.3%, *χ*^2^ = 6.67, *P* = 0.010, 95%CI:9.7%–43.3%). The study group reported 6.7% mild gastrointestinal discomfort, with no serious adverse events.

**Conclusion:**

As an add-on therapy to psychological counseling, Sini San combined with Xiaoxianxiong Decoction shows preliminary short-term efficacy in alleviating depressive symptoms and improving quality of life in adolescents with target TCM syndrome, with favorable short-term safety. Due to small sample size, single-center design, lack of double-blinding and placebo control, the findings are hypothesis-generating and cannot support broad clinical promotion.

## 1 Introduction

Adolescence is a critical period of physical and psychological development, and adolescent depression has become a globally prevalent mental health issue (1, 2). Characterized by persistent low mood, irritability, anhedonia, and sleep/appetite disturbances, it impairs cognitive development, emotional regulation, academic performance, and social adaptability, and may even lead to extreme behaviors in severe cases. Its incidence is rising annually due to increased academic and life stressors, attracting widespread attention in the medical field (3). Current clinical management of adolescent depression encompasses psychological interventions and pharmacotherapy (4, 5) Psychological counseling, as a foundational intervention, helps adolescents identify and correct negative cognitive patterns and alleviate emotional distress. Notably, adolescents have immature physical and psychological development, making them more vulnerable to adverse reactions from conventional pharmacotherapy and requiring more targeted therapeutic strategies. However, monotherapy with psychological counseling has limited efficacy, particularly in moderate-to-severe cases, and exhibits a slow onset of action.

Traditional Chinese medicine (TCM, Bian Zheng Lun Zhi), emphasizing syndrome differentiation and treatment and holistic regulation of the body's functional state, offers unique advantages in improving symptoms and minimizing adverse reactions—making it well-suited for adolescents with immature physical development. In traditional Chinese medicine (TCM) theory, adolescent depression belongs to the category of “Yu Zheng” (melancholia) (6). Due to adolescents' unique physiological characteristics (vigorous yang-qi, unstable emotions, and immature spleen-stomach function), its pathogenesis is mainly manifested as liver qi stagnation and phlegm-heat blocking the middle energizer. Long-term emotional suppression causes liver qi stagnation, which accumulates heat and disturbs the mind, while improper diet impairs spleen function, leading to endogenous phlegm-dampness that binds with heat and exacerbates depression. Therefore, the core therapeutic principles are soothing the liver to clear heat, resolving phlegm, and relieving stuffiness. Sini San, a classic TCM formula from Treatise on Febrile Diseases, comprises *Bupleuri Radix*, *Aurantii Fructus Immaturus*, *Paeoniae Radix Alba*, and *Glycyrrhizae Radix et Rhizoma*. It exerts effects of soothing the liver to relieve stagnation and regulating qi to alleviate pain (7). Xiaoxianxiong Tang, also from Treatise on Febrile Diseases, consists of *Coptidis Rhizoma, Pinelliae Rhizoma Praeparatum*, and *Trichosanthis Fructus* (the dried ripe fruit of *Trichosanthes kirilowii Maxim*.), with functions of clearing phlegm-heat, resolving masses, and relieving stuffiness. The combination of these two formulas synergistically soothes the liver, clears heat, resolves phlegm, and relieves stuffiness, directly targeting the pathogenesis of adolescent depression with the aforementioned syndrome.

To date, no clinical studies have specifically evaluated the efficacy and safety of Sini San combined with Xiaoxianxiong Tang in this patient population—despite adolescents' unique physiological vulnerability. This randomized controlled study, with psychological counseling as the control, adopts an add-on therapy design to investigate the short-term clinical efficacy and safety of this combined TCM formula, aiming to provide preliminary targeted evidence for TCM syndrome-based treatment of adolescent depression with this specific syndrome type.

## 2 Methods

### 2.1 General information

A total of 60 adolescents with depression who met the inclusion criteria were recruited from the Pediatrics Department of Youxian Branch of The Third Hospital of Mianyang (Sichuan Mental Health Center/The Affiliated Mianyang Hospital of Chongqing Medical University) between January 2024 and December 2025. Randomization sequence generation was conducted using a computer-generated random number table via SPSS 26.0 software by an independent statistician not involved in the trial implementation and outcome assessment. Allocation concealment was achieved by sealing the grouping results in sequentially numbered opaque envelopes, which were opened only after the participant completed baseline assessment and signed the informed consent form. Finally, participants were randomly assigned in a 1:1 ratio to the study group (TCM decoction+psychological intervention) or the control group (psychological intervention alone), with 30 cases in each group.

In the study group, there were 16 males and 14 females, aged 12–18 years, with an average age of (15.12 ± 1.78) years, and a course of disease of 2–16 months, with an average course of (8.95 ± 3.12) months. In the control group, there were 15 males and 15 females, aged 12–17 years, with an average age of (14.96 ± 1.83) years, and a course of disease of 2–18 months, with an average course of (9.13 ± 3.25) months. There were no statistically significant differences in gender, age, course of disease, CDRS-R score, TCM syndrome score, or PedsQL score between the two groups before treatment (all *P* > 0.05), indicating comparability. All participants had moderate depression at baseline, with mean CDRS-R scores of 28.76 ± 3.12 in the study group and 28.54 ± 3.21 in the control group (*P* = 0.79)

**Figure 1:**
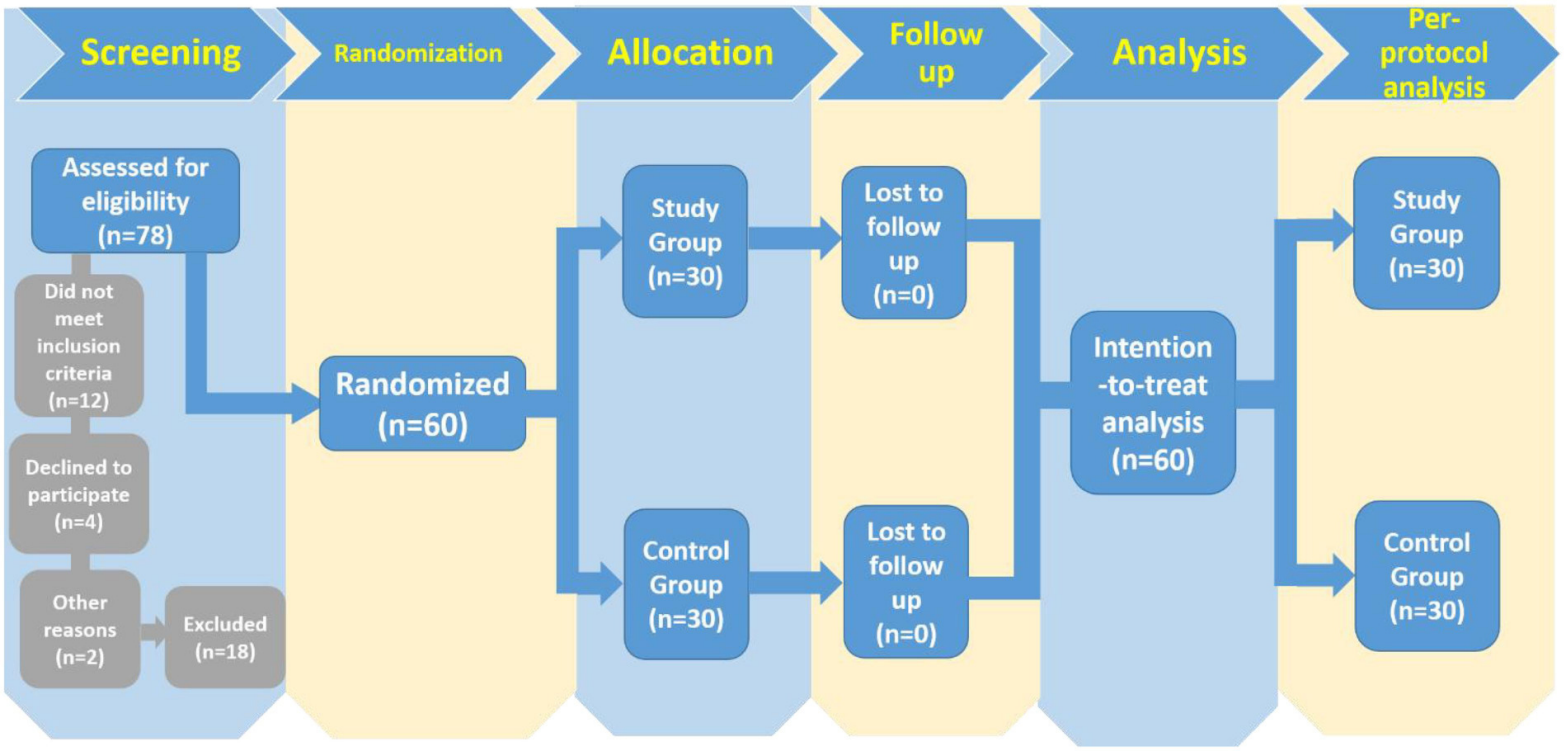
The consort flow diagram of the trial.

A total of 78 adolescents were initially assessed for eligibility between January 2024 and December 2025 at the Pediatrics Department of Youxian Branch of The Third Hospital of Mianyang. Eighteen participants were excluded for the following reasons: 12 did not meet the predefined inclusion criteria, 4 declined to participate, and 2 were excluded for other reasons. Finally, 60 eligible participants were randomly assigned in a 1:1 ratio to the study group (*n* = 30) or the control group (*n* = 30). All participants completed the full 8-week intervention and follow-up without any loss to follow-up or intervention discontinuation. The participant flow through all stages of this randomized controlled trial is shown in (Figure 1).

This diagram summarizes the number of participants at each stage of the trial, including screening, randomization, allocation, intervention, follow-up, and final analysis.

### 2.2 Inclusion and exclusion criteria

#### 2.2.1 Inclusion criteria

① Meet the diagnostic criteria for depression in the Diagnostic and Statistical Manual of Mental Disorders, 5th Edition (DSM-5) (1); ② TCM syndrome differentiation conforms to the syndrome of liver qi stagnation transforming into heat and phlegm-heat stagnation in the chest, with main symptoms including low mood, irritability and easy anger, chest tightness and stuffiness; secondary symptoms including bitter mouth and dry throat, poor appetite, insomnia and dreaminess, red tongue with yellow and greasy coating, stringy and rapid pulse (8); Clinical Application Guidelines for Chinese Patent Medicines in the Treatment of Depressive Disorder (9) ③ Aged 12–18 years; ④ CDRS-R score ≥20 points at baseline (indicating moderate depressive severity); ⑤ No use of antidepressant drugs, TCM decoctions, or other mental intervention measures in the past 1 month; ⑥ Voluntarily participate in the study and cooperate with follow-up.

#### 2.2.2 Exclusion criteria

① Complicated with other mental diseases such as schizophrenia, bipolar affective disorder, and autism spectrum disorder; ② Complicated with severe liver, kidney, heart, or other organ dysfunction; ③ Complicated with immune system diseases, endocrine diseases, or malignant tumors; ④ Allergic to the Chinese medicinal materials used in the study; ⑤ Poor compliance, unable to complete the intervention course or follow-up; ⑥ With a history of drug abuse or alcoholism.

### 2.3 Intervention methods

#### 2.3.1 Control group

Participants received psychological counseling alone, with a unified intervention plan: ① Cognitive behavioral intervention: guide adolescents to recognize and correct negative cognitive tendencies, 45 min per session; ② Family support guidance: communicate with guardians, guide scientific parenting methods, and assist in improving the family support system, 30 min per session. The above psychological counseling was conducted once a week for 8 consecutive weeks.

#### 2.3.2 Study group

On the basis of the same psychological counseling as the control group, Sini San combined with Xiaoxianxiong Tang was added for treatment. This study adopts an add-on therapy design, and thus cannot evaluate the standalone efficacy of the TCM decoction alone for adolescent depression.

##### 2.3.2.1 Prescription composition

Bupleuri Radix 15 g, Aurantii Fructus Immaturus 15 g, Paeoniae Radix Alba 15 g, Glycyrrhizae Radix et Rhizoma 6 g, Coptidis Rhizoma 6 g, Pinelliae Rhizoma Preparatum 10 g, Trichosanthis Fructus 15 g. For adolescents aged 12–14 years, the dosage should be reduced by half; for those aged 15–18 years, the full dosage is administered.

All Chinese medicinal materials were purchased from the Chinese Medicinal Decoction Piece Room of Youxian Branch of The Third Hospital of Mianyang, complying with the standards of the Pharmacopoeia of the People's Republic of China (18). All medicinal materials were authenticated by Director Gu Xuegang (Department of Clinical Pharmacy, The Third Hospital of Mianyang) based on the aforementioned pharmacopoeia standards. Voucher specimens (No. SNS-202401 for Sini San; XXXT-202401 for Xiaoxianxiong Tang) were deposited in the Specimen Repository of the Department of Clinical Pharmacy, Youxian Branch of The Third Hospital of Mianyang, for future reference.

##### 2.3.2.2 Decocting method

soak the medicinal materials in 500 mL water for 30 min, boil with high heat, then decoct with low heat for 20 min, filter to obtain 200 mL decoction; add 300 mL water again, decoct for 15 min, filter to obtain 150 mL decoction; mix the two decoctions (total 350 mL), take orally in two portions (175 mL each) 30 min after breakfast and dinner, 1 dose per day, for 8 consecutive weeks. Psychological counseling was the same as that of the control group. All decoctions were prepared by a single trained pharmacist in the hospital pharmacy using standardized equipment and protocols to ensure consistency of the intervention.

### 2.4 Observation indicators

Primary Outcomes (assessed at baseline, 4 weeks, and 8 weeks):

Children's Depression Rating Scale-Revised (CDRS-R) (10) score, measuring the severity of depressive symptoms.

TCM syndrome score, a commonly used indicator for assessing the therapeutic efficacy of TCM, was employed for measuring the improvement of TCM-specific manifestations (11).

#### 2.4.1 Secondary outcomes

Pediatric Quality of Life Inventory (PedsQL) score (assessed at baseline and 8 weeks), measuring health-related quality of life.

Clinical effective rate (assessed at 8 weeks), based on CDRS-R score reduction and TCM syndrome improvement.

Adverse events (monitored weekly throughout the intervention), evaluating safety.

All outcome assessments were performed by two independent researchers who were blinded to the grouping of participants. If there was a discrepancy in the scores (difference >3 points for CDRS-R or >2 points for TCM syndrome score), a third senior researcher was invited to make a consensus judgment. It should be noted that participants and clinicians could not be blinded due to the oral administration of TCM decoction in the study group, which may introduce performance bias and expectancy/placebo effects.

① Children's Depression Rating Scale-Revised (CDRS-R) (10): Evaluate the severity of depressive symptoms at baseline, 4 weeks, and 8 weeks post-treatment. The scale includes 17 items, with a total score of 0–60 points. The higher the score, the more severe the depressive symptoms. ② TCM syndrome score (12): Developed and standardized by the National Medical Products Administration of China in the Guidelines for Clinical Application Guidelines for Chinese Patent Medicines in the Treatment of Depressive Disorder (9) this scale is the most widely used tool for evaluating TCM syndrome outcomes in depression clinical trials in China. Main symptoms (low mood, irritability and easy anger, chest tightness and stuffiness) are scored 0–3 points (0 points for no symptoms, 1 point for mild, 2 points for moderate, 3 points for severe); secondary symptoms (bitter mouth and dry throat, poor appetite, insomnia and dreaminess) are scored 0–2 points (0 points for no symptoms, 1 point for mild, 2 points for moderate). The total score is the sum of main and secondary symptom scores, and the lower the score, the better the improvement of TCM syndrome. ③ Pediatric Quality of Life Inventory (PedsQL) (13): Evaluate the quality of life at baseline and 8 weeks post-treatment, including physical function, emotional function, social function, and school function, with a total score of 0–100 points. The higher the score, the better the quality of life. ④ Clinical efficacy: Evaluated at 8 weeks post-treatment, referring to the Clinical Application Guidelines for Chinese Patent Medicines in the Treatment of Depressive Disorder (9). Cure: CDRS-R score decreased by ≥75%, TCM syndrome symptoms basically disappeared; Marked effect: CDRS-R score decreased by 50%–74%, TCM syndrome symptoms significantly improved; Effective: CDRS-R score decreased by 25%–49%, TCM syndrome symptoms improved; Ineffective: CDRS-R score decreased by <25%, TCM syndrome symptoms did not improve or worsen. Total effective rate=(number of cured cases+number of marked effective cases+number of effective cases)/total number of cases ×100%. ⑤ Adverse events: A structured weekly follow-up protocol (face-to-face or telephone interview) and routine laboratory tests (liver/kidney function, blood routine) at baseline and post-intervention were adopted to monitor adverse events. Adverse events were graded according to the Common Terminology Criteria for Adverse Events (CTCAE) v5.0 (14). Record the occurrence of adverse events and their severity, duration, and clinical management.

### 2.5 Statistical methods

SPSS 26.0 statistical software was used for data analysis. Normality of measurement data was tested using the Shapiro–Wilk test. Multiple t-tests with Bonferroni correction (paired t-test for intragroup comparison, independent sample t-test for intergroup comparison) were used for short-term outcome analysis, and a sensitivity analysis using repeated-measures ANOVA confirmed the consistency of results. Given the limited number of 3 time points (baseline, 4 weeks, 8 weeks) and the clear *a priori* hypotheses for each time-point comparison, multiple t-tests with Bonferroni correction were considered appropriate and more clinically interpretable for readers. This analytic strategy was selected over repeated-measures ANOVA to enable straightforward, time-specific reporting of within-group and between-group differences, which better highlights the clinically meaningful short-term efficacy changes at each scheduled assessment. Measurement data conforming to normal distribution were expressed as (x ± s), paired t-test was used for intragroup comparison before and after treatment, and independent sample t-test was used for intergroup comparison; measurement data not conforming to normal distribution were expressed as median (interquartile range), and Wilcoxon rank sum test was used. Count data were expressed as n(%), and *χ*^2^ test was used. Effect sizes (Cohen's d) and 95% confidence intervals (CIs) were calculated for all key outcomes. Bonferroni correction was applied for multiple comparisons, with the adjusted *α* level set at 0.017. Intention-to-treat (ITT) analysis was performed for all randomized participants, and the last observation carried forward (LOCF) method was used for missing data. *P* < 0.017 was considered statistically significant.

## 3 Results

### 3.1 Comparison of CDRS-R scores between two groups

**Figure 2:**
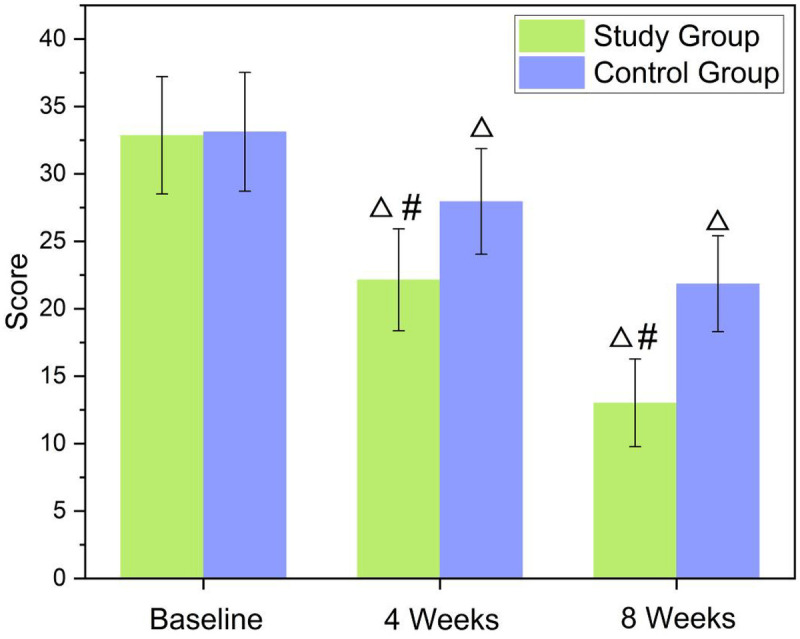
Comparison of CDRS-R scores between two groups at baseline, 4 weeks, and 8 weeks post-treatment. Data are expressed as mean ± SD; △*P* < 0.001 vs. baseline, #*P* < 0.001 vs. control group; Error bars represent standard deviations (SD).

At baseline, there was no statistically significant difference in CDRS-R score between the two groups (*P* > 0.05). After 4 and 8 weeks of intervention, the CDRS-R scores of both groups were significantly lower than those at baseline (all *P* < 0.001); at the same time points, the CDRS-R score of the study group was significantly lower than that of the control group (all *P* < 0.001). At 4 weeks, Cohen's d = 1.21, 95%CI: 0.52–1.90; at 8 weeks, Cohen's d = 2.64, 95%CI: 1.72–3.56 See Figure 2.

### 3.2 Comparison of TCM syndrome scores between tswo groups

**Figure 3:**
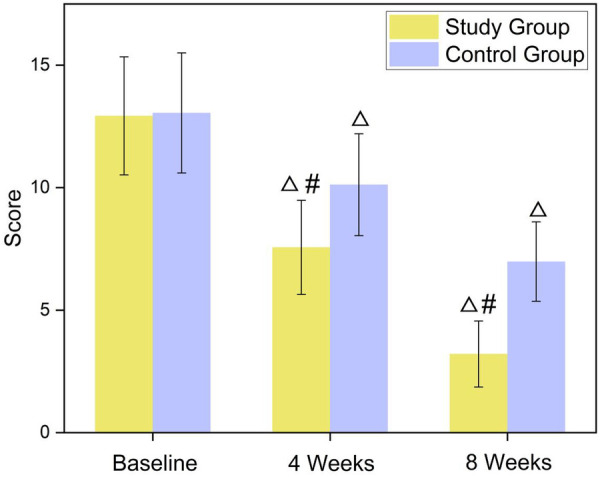
Comparison of TCM syndrome scores between two groups at baseline, 4 weeks, and 8 weeks post-treatment. Data are expressed as mean ± SD; △*P* < 0.001 vs. baseline, #*P* < 0.001 vs. control group; Error bars represent standard deviations (SD).

At baseline, there was no statistically significant difference in TCM syndrome score between the two groups (*P* > 0.05). After 4 and 8 weeks of intervention, the TCM syndrome scores of both groups were significantly lower than those at baseline (all *P* < 0.001); at the same time points, the TCM syndrome score of the study group was significantly lower than that of the control group (all *P* < 0.001). At 4 weeks, Cohen's d = 1.15, 95%CI:0.47–1.83; at 8 weeks, Cohen's d = 2.52, 95%CI:1.61–3.43 See Figure 3.

### 3.3 Comparison of PedsQL scores between two groups

**Figure 4:**
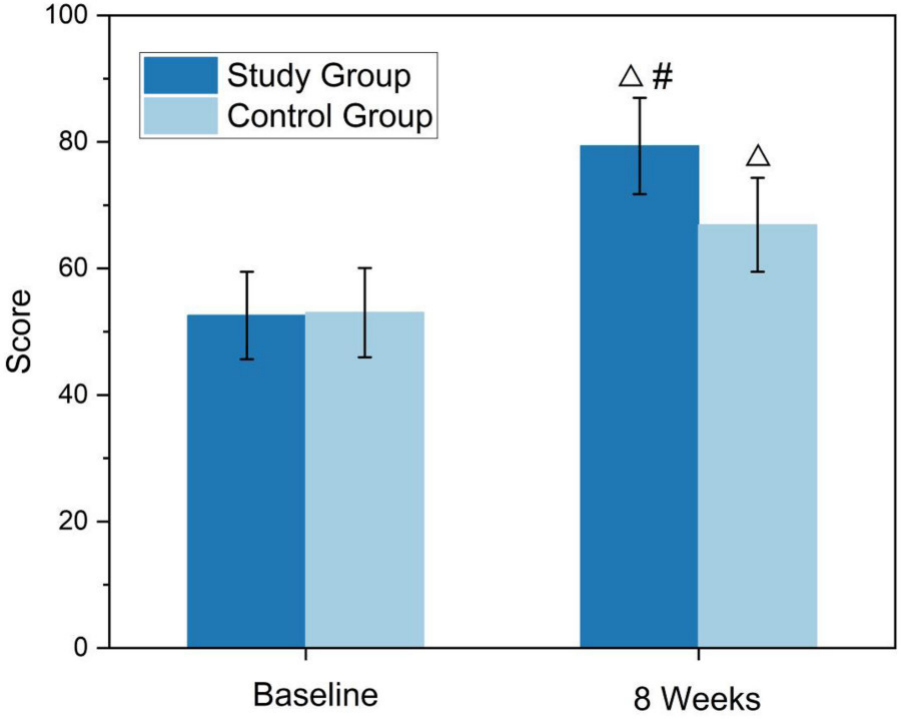
Comparison of PedsQL scores between two groups at baseline and 8 weeks post-treatment. Data are expressed as mean ± SD; △*P* < 0.001 vs. baseline, #*P* < 0.001 vs. control group; Error bars represent standard deviations (SD).

At baseline, there was no statistically significant difference in PedsQL score between the two groups (*P* > 0.05). After 8 weeks of intervention, the PedsQL scores of both groups were significantly higher than those at baseline (all *P* < 0.001); the PedsQL score of the study group was significantly higher than that of the control group (*P* < 0.001). Cohen's d = 1.68, 95%CI:0.92–2.44 See Figure 4.

### 3.4 Comparison of clinical efficacy between two groups

**Figure 5:**
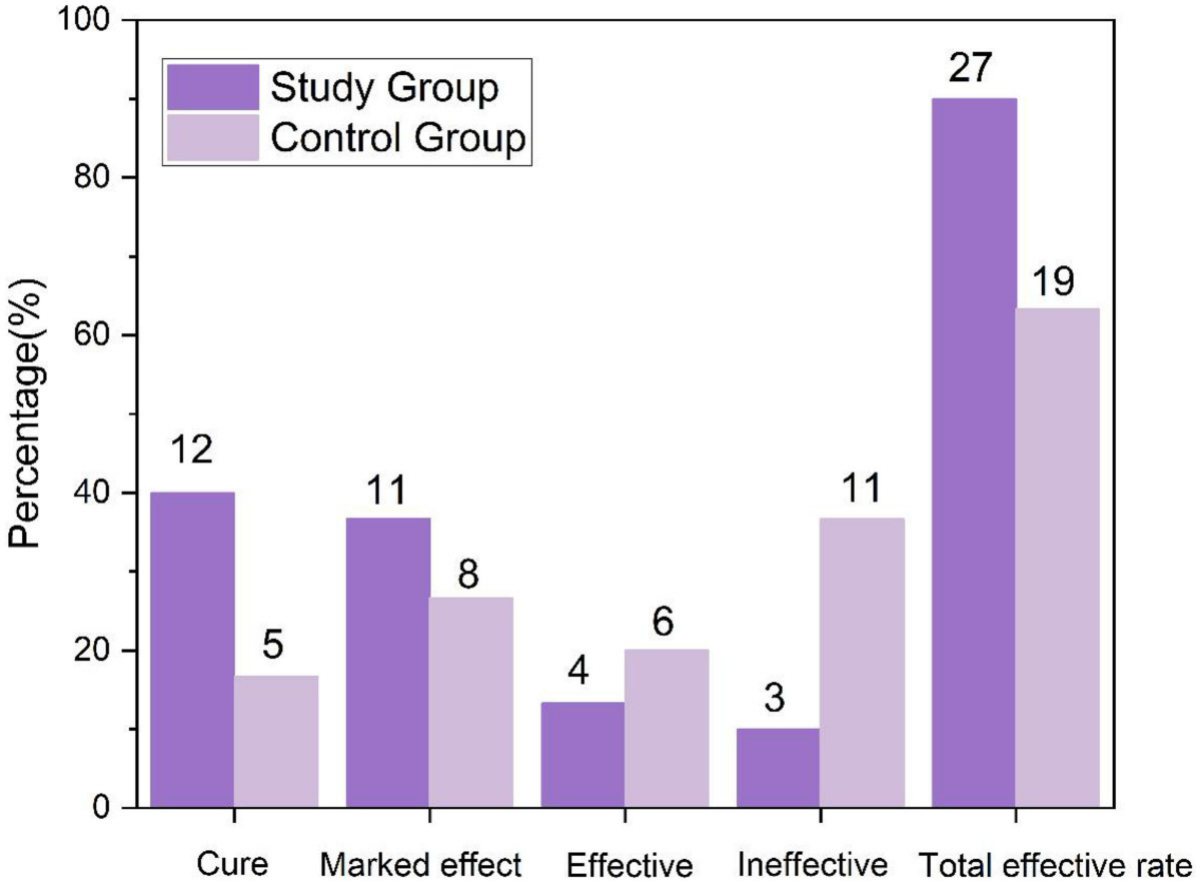
Comparison of clinical efficacy between two groups (n, %). The bar chart shows the number of patients in each efficacy category. The “Total effective rate” bar displays the number of patients with “Cure+Marked effect+Effective” in each group (27 in the study group, 19 in the control group), corresponding to the total effective rate of 90.0% and 63.3%, respectively.

After 8 weeks of intervention, the total clinical effective rate of the study group was 90.00% (27/30), which was significantly higher than 63.33% (19/30) of the control group (*χ*^2^ = 6.67, *P* = 0.010, 95%CI:9.7%–43.3%) See Figure 5.

### 3.5 Comparison of adverse reactions between two groups

**Figure 6:**
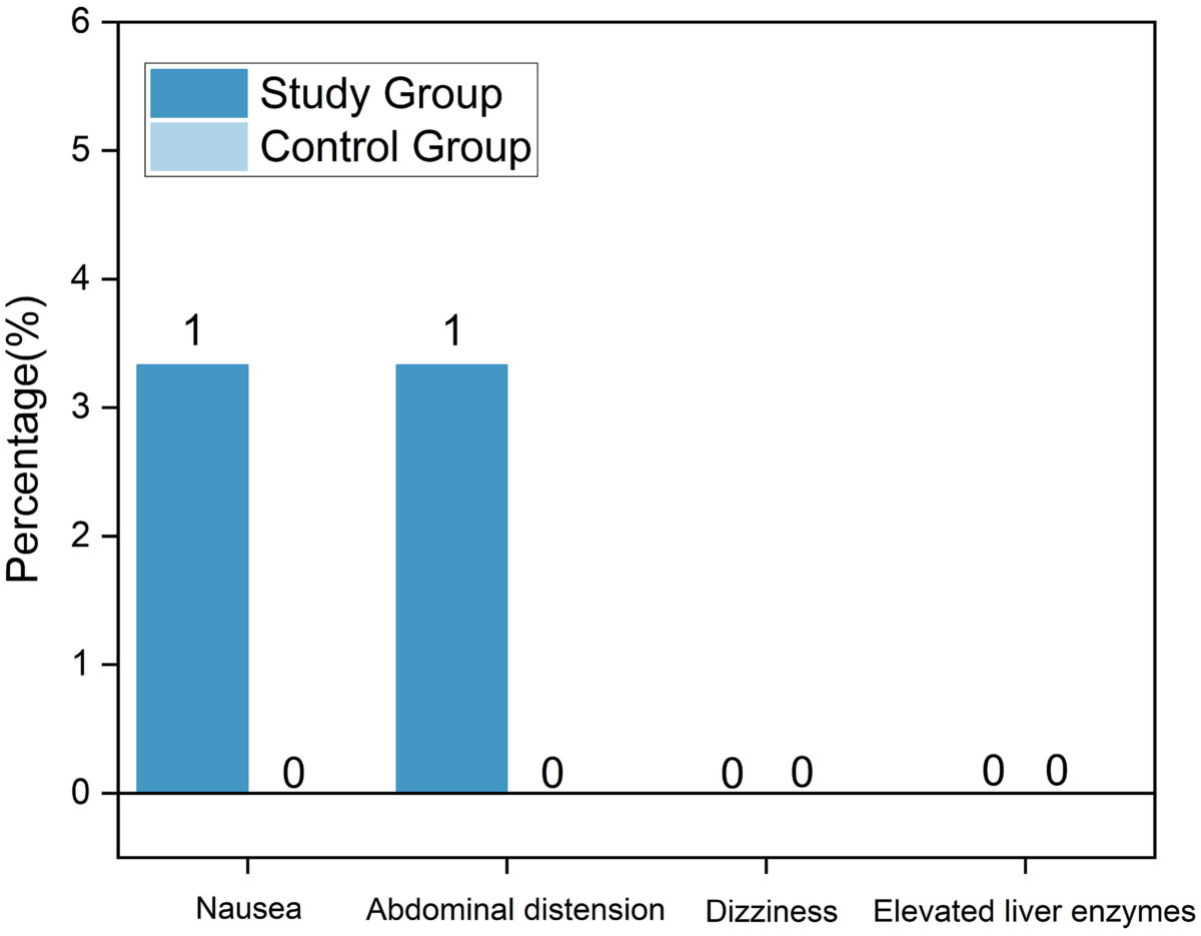
Comparison of adverse event incidence between two groups (%).

During the intervention period, no adverse events occurred in the control group. In the study group, 2 cases had mild adverse events, with an incidence rate of 6.67%, all of which were mild nausea and abdominal distension. After adjusting the medication time to 1 h post-meal, the symptoms improved, and no serious adverse events occurred. After treatment, the liver and kidney function and blood routine of the two groups were normal, with no abnormal changes See Figure 6.

## 4 Discussion

Adolescent depression is a complex mental disease induced by multiple factors, involving biological, psychological, and social factors. Psychological counseling, as a basic intervention method, can help adolescents adjust their cognitive state and alleviate emotional pressure (4), but its single intervention is difficult to achieve an ideal therapeutic effect, especially for patients with moderate to severe depression. TCM treats from the holistic concept, combines syndrome differentiation and treatment, and has obvious advantages in improving symptoms and reducing adverse reactions, which has important clinical significance for the treatment of adolescent depression (8).

From the perspective of TCM, the occurrence of adolescent depression is closely related to the dysfunction of zang-fu organs such as the liver and spleen. Adolescents have vigorous emotions, and long-term depression leads to stagnation of liver qi, which fails to dredge and disperse, and turns into heat after a long time, disturbing the mind, resulting in low mood, irritability, and other symptoms; at the same time, adolescents' spleen and stomach function is not perfect, improper diet leads to dysfunction of spleen transportation, endogenous phlegm-dampness, binding of phlegm and heat, blocking the chest and middle jiao, resulting in chest tightness, poor appetite, and other manifestations, which further affects emotional regulation. Therefore, soothing the liver and clearing heat, resolving phlegm and relieving stuffiness are the key to treatment.

Sini San is a classic prescription for soothing the liver and relieving stagnation. Bupleuri Radix is the monarch drug, which can soothe the liver and relieve stagnation, dredge the meridians and collaterals; Aurantii Fructus Immaturus is the minister drug, which breaks qi and relieves stagnation, eliminates distension and stuffiness, and assists Bupleuri Radix to enhance the effect of regulating qi; Paeoniae Radix Alba nourishes blood and softens the liver, relieves spasm and pain, and prevents Bupleuri Radix from being too dry to damage yin; Glycyrrhizae Radix et Rhizoma Praeparata cum Melle tonifies qi and harmonizes the middle energizer, and harmonizes all drugs (15).

Xiaoxianxiong Decoction is aimed at phlegm-heat stagnation in the chest. Coptidis Rhizoma clears away heat and dries dampness, purges fire and detoxifies; Pinelliae Rhizoma Praeparatum dries dampness and resolves phlegm, reduces adverse flow and relieves stuffiness; Trichosanthis Fructus clears away heat and resolves phlegm, relieves stuffiness and eliminates masses. The three drugs cooperate to clear away phlegm-heat and open the chest to relieve stuffiness.

Modern pharmacological studies have shown that Bupleuri Radix can regulate the levels of neurotransmitters such as serotonin and dopamine in the brain (16), while Coptidis Rhizoma inhibits neuroinflammation by downregulating the TLR4/NF-*κ*B pathway (17). Notably, these two studies were published online during the revision of this manuscript and provide the latest mechanistic evidence for our findings. All mechanistic explanations are based on preclinical and previous clinical studies and remain purely hypothetical, as no biomarker analyses (e.g., serum neurotransmitter levels or inflammatory markers) were performed in the current trial.These potential mechanisms may synergistically contribute to the reduction of depressive symptoms observed in the study group, which is consistent with the significant decrease in CDRS-R scores.The combination of the two prescriptions can soothe the liver and clear heat, resolve phlegm and relieve stuffiness, target the pathogenesis of adolescent depression with liver qi stagnation transforming into heat and phlegm-heat stagnation in the chest, may achieve the effect of alleviating both depressive symptoms and TCM syndrome manifestation.The results of this study show that after 4 and 8 weeks of intervention, the CDRS-R scores and TCM syndrome scores of both groups are lower than those at baseline, and the PedsQL scores are higher than those at baseline, indicating that both psychological counseling alone and Sini San combined with Xiaoxianxiong Tang plus psychological counseling can alleviate depressive symptoms, improve TCM syndrome manifestations and quality of life of adolescent depression patients. At the same time points, the CDRS-R score and TCM syndrome score of the study group are lower than those of the control group, and the PedsQL score is higher than that of the control group, suggesting that Sini San combined with Xiaoxianxiong Tang can enhance the intervention effect and better improve the patient's condition.

The total clinical effective rate of the study group is 90.00%, which is significantly higher than 63.33% of the control group, indicating that the combined intervention of TCM decoction and psychological counseling has higher clinical efficacy. In terms of safety, no adverse events occurred in the control group, and only 2 cases in the study group have mild gastrointestinal discomfort, which improves after adjusting the medication time, and no serious adverse events occur, indicating that Sini San combined with Xiaoxianxiong Tang has good safety and high clinical applicability for adolescents.

The observed effect sizes (Cohen's d = 1.15–2.64) are substantially larger than the 0.3–0.8 range typically reported in general adolescent depression trials. This discrepancy is not unexpected and can be explained by three key study design features. First, we adopted a strict syndrome-specific inclusion criterion, enrolling only patients with Liver Qi Stagnation and Phlegm-Heat Obstruction syndrome—the exact subtype targeted by our TCM formula. This targeted approach excluded patients with other TCM syndromes who would likely show a weaker response, thus amplifying the observed treatment effect. Second, our add-on design measures the incremental benefit of TCM over standard psychological counseling, not the standalone effect of either intervention. Third, as a pilot single-center RCT with a small sample size, effect size overestimation is a well-documented methodological limitation, which we explicitly acknowledge in our limitations section.

## 5 Limitations

This study has several important limitations that should be acknowledged:

Single-center design and small sample size (*n* = 60), which reduces the external validity of the findings, limits statistical power to detect smaller effect sizes, and may lead to overestimation of the true treatment effect.

Lack of double-blinding and placebo control. The intervention included TCM herbal medicine vs. psychological counseling alone, resulting in the absence of a placebo control. Due to the nature of oral TCM decoction administration, participants and treating clinicians could not be blinded, which may introduce performance bias and placebo effects on subjective outcome measures. Previous TCM clinical studies also adopted a design of Chinese herbal medicine combined with Western medicine vs. Western medicine alone without a placebo control group, which was attributed to the difficulty in preparing TCM decoctions with consistent appearance, taste, and smell for valid blinding (2). A placebo-controlled design was not implemented due to challenges in preparing a visually and organoleptically identical TCM placebo that would maintain blinding in this adolescent population.

Add-on therapy design, which prevents evaluation of the standalone efficacy of Sini San combined with Xiaoxianxiong Tang for adolescent depression.

Short intervention duration (8 weeks) with no long-term follow-up, so the sustained efficacy and recurrence rates of the intervention could not be assessed.

Reliance on subjective outcome measures, which are susceptible to judgment bias despite the use of blinded outcome assessors.In addition, the TCM syndrome score, while validated in Chinese practice, is not widely recognized internationally, limiting generalizability to non-Chinese populations.

No biomarker detection was performed, so the underlying molecular mechanisms of the intervention remain speculative.

Limited safety assessment, as the small sample size and short follow-up period prevent detection of rare or delayed adverse events.

In the future, multi-center, large-sample, and long-term follow-up studies can be carried out to further verify the efficacy and safety of the prescription, and in-depth exploration of its mechanism of action can be conducted to provide more reliable evidence for clinical application.

In conclusion, preliminary evidence from this small-sample, single-center RCT suggests that Sini San combined with Xiaoxianxiong Tang as an add-on therapy to psychological counseling has significant short-term efficacy in the treatment of adolescent depression with the syndrome of liver qi stagnation and phlegm-heat blocking the middle energizer. It can effectively alleviate depressive symptoms, improve TCM syndrome manifestations and quality of life, with favorable short-term safety. However, due to the study's limitations, the findings are hypothesis-generating and cannot support broad clinical promotion. Future multi-center, large-sample, double-blinded placebo-controlled trials with long-term follow-up are needed to confirm these results.

## Data Availability

The original data supporting the findings of this study are available from the Corresponding author upon reasonable request.
